# Eating Disorders in Youth with Chronic Health Conditions: Clinical Strategies for Early Recognition and Prevention

**DOI:** 10.3390/nu15173672

**Published:** 2023-08-22

**Authors:** Maya Michelle Kumar

**Affiliations:** Division of Adolescent and Young Adult Medicine, Department of Pediatrics, University of California San Diego, San Diego, CA 92123, USA; m8kumar@health.ucsd.edu

**Keywords:** adolescent, chronic disease, feeding and eating disorders, mental health, primary prevention

## Abstract

Youth with chronic health conditions face an elevated risk of eating disorders and disordered eating behaviors. Contributors to this phenomenon may include the unique threats faced by this vulnerable population to their body image, their relationships with food and eating, and their mental health and self-esteem. However, youth with chronic health conditions may also experience more severe medical complications and mortality from eating disorder behaviors because of the additional risks conveyed by their underlying conditions. In this review, clinical strategies are provided to support youth with chronic health conditions through early recognition of eating disorder behaviors and prompt referral to treatment, which is important for a better prognosis. Suggestions are also given to mitigate their risk of developing eating disorders by proactively addressing risk factors and offering thoughtful anticipatory guidance that promotes a positive relationship with food and eating.

## 1. Introduction

Eating disorders refer to psychiatric conditions upon which the primary effect is on eating or feeding. While some struggles with body image may be a normal part of adolescent development, eating disorders encompass thoughts and behaviors that significantly impair an individual’s physical and psychosocial functioning. The Diagnostic and Statistical Manual of Mental Disorders, 5th Edition (DSM-5), describes four major eating disorders: anorexia nervosa, bulimia nervosa, avoidant–restrictive food intake disorder (ARFID), and binge-eating disorder [1]. Eating disorders are highly prevalent among young people. A recent review found that in Western nations, the lifetime prevalence of DSM-5-diagnosable eating disorders (anorexia nervosa, bulimia nervosa, binge-eating disorder, and other specified feeding and eating disorders) ranged from 5.5 to 17.9% among young women and 0.6 to 2.4% among young men, with emerging data from Eastern Europe, Latin America, and Asia showing a similarly high prevalence [2]. These studies are likely underestimates, as they did not include individuals reporting ARFID. Since the start of the COVID-19 pandemic, the incidence and severity of eating disorders has increased globally, especially among children and adolescents [3]. Eating disorders, particularly anorexia nervosa, have one of the highest mortality rates of any psychiatric illness [4].

Studies have identified many risk factors for the development of eating disorders, including genetic predisposition, high-risk personality traits, comorbid mental health conditions, family dynamics, social and peer influences, differences in the immune and endocrinological systems, differences in gut microbiota, and in utero exposures [5,6,7,8,9]. Unfortunately, there is evidence that having a chronic health condition, including physical and intellectual disabilities, is another important risk factor in the development of eating disorders and disordered eating. Compared with their peers, adolescents with chronic health conditions are more likely to actively try to lose weight and engage in unhealthy weight control behaviors (e.g., fasting, self-induced vomiting, or the use of diet pills or laxatives) [10,11,12,13,14]. Chronic health conditions are also strongly associated with ARFID [15,16,17,18,19,20] and binge-eating disorder [21,22,23,24]. However, youth with chronic health conditions may also experience more severe medical complications and mortality with eating disorder behaviors than their peers because of the additional risks conveyed by their underlying conditions [23,24,25,26,27,28,29,30]. This is an illustration of the “double whammy” phenomenon described by Sawyer et al. [31]: youth with chronic health conditions are doubly disadvantaged, because compared with their peers, they are both more likely to engage in risk behaviors and more likely to suffer serious consequences from those behaviors.

## 2. Risk Factors for Body-Image-Related Disordered Eating Behaviors

There are many factors which may place youth with chronic health conditions at higher risk for developing body-image-related eating disorders.

### 2.1. Impact of Chronic Conditions on Body Image

Developing acceptance of one’s weight and/or shape is already a challenging aspect of normal adolescent development, but studies have shown that adolescents with chronic health conditions have poorer body image and higher body dissatisfaction than their peers [10,11,32,33,34,35,36]. Chronic health conditions, and/or the treatments they require, often lead to short stature, delayed puberty, altered body composition, use of assistive devices or medical hardware, or other significant alterations in physical appearance [37]. They may be associated with visible congenital anomalies or dysmorphic physical features. Patients may require treatments that cause weight gain, such as steroids, antipsychotics, or glucose-lowering medications. All of these may significantly impair body image and self-esteem beyond what would be developmentally normal for this age group, greatly increasing the risk for body-image-based eating disorders such as anorexia nervosa and bulimia nervosa and disordered eating behaviors. 

### 2.2. Increased Focus on Diet as Part of Disease Management

Youth who require dietary treatment for their chronic health conditions are at particularly high risk for body-image-based eating disorders. Being taught by health care providers and caregivers to pay attention to nutrition labels, food ingredients, and eating and exercise patterns in order to stay healthy, and having one’s weight monitored far more closely than healthy peers, can draw excessive attention to food and weight that may evolve into disordered eating behaviors or a clinical eating disorder. As such, a higher prevalence of body-image-related eating disorders and disordered eating behaviors have been particularly observed among youth with type I diabetes mellitus [38], celiac disease [39], cystic fibrosis [25,40], inflammatory bowel disease [41], food allergies [42], and inborn errors of metabolism [43]. 

### 2.3. Higher Risk of Anxiety, Depression, and Stress

Among adolescents and young adults, the presence of anxiety and depression may be initiating and/or maintaining factors for eating disorder symptomatology [44,45,46,47]. Multiple types of disordered eating behaviors, such as dietary restriction, purging, and binge-eating, are frequently used as coping mechanisms in response to stress, negative emotions (including sadness, anger, and fear), and poor self-efficacy [48,49,50,51]. Co-occurring psychiatric conditions are also known to worsen eating disorder outcomes [4]. Unfortunately, youth with chronic health conditions are at much higher risk for depression, anxiety, stress, fear, poor self-esteem, and other negative emotions than their peers [36,52,53,54,55], making it more likely that they would engage in eating disorder behaviors. 

## 3. Risk Factors for ARFID

Adolescents and young adults with chronic health conditions may be particularly predisposed to ARFID [15,16,17,18,19,20]. In fact, one study reported that over 50% of youth with ARFID have a comorbid medical condition [18]. ARFID is an eating disorder that is unrelated to body image but, rather, stems from other mental or behavioral health issues, while the primary effect remains on feeding and eating [1]. These may include fear of adverse reactions from eating (e.g., choking, vomiting, allergic reaction), extremely picky eating that significantly limits intake, or poor appetite cues with little intrinsic motivation to seek out food [56,57]. Though the psychopathology is different, patients with ARFID may experience medical complications of the same severity as those with AN [58,59]. They may also experience a substantial reduction in psychosocial functioning, such as an inability to eat in social situations or outside of their homes, dependency on the use of liquid supplements, or dependence on artificial enteric or non-enteric nutritional supplementation [1]. 

Youth with chronic health conditions may have a long history of failure to thrive in earlier childhood, which may ‘normalize’ the state of being thin and small even if their underlying condition is now well-controlled. They may have experienced abdominal pain, poor appetite, diarrhea, nausea, or vomiting as symptoms of their underlying condition (e.g., inflammatory bowel disease, celiac disease) or side effects of the treatments they required (e.g., chemotherapy), which may lead to a fear of re-experiencing these symptoms with eating, lack of interest in food, negative associations with food, and/or extreme pickiness. Studies have shown that youth with chronic health conditions may continue to avoid foods even after the resolution of their disease-related symptoms [16]. Some youth may fear having to frequently use the bathroom or empty an ostomy bag after eating, especially when at school or in social situations, which may lead them to intentionally skip meals when outside of the home. 

As previously mentioned, youth with chronic health conditions have a high prevalence of anxiety, which is strongly associated with ARFID [20,58]. Autism spectrum disorder and other neurodevelopmental disorders also appear to be significant risk factors for ARFID [60]. Of concern, despite their elevated risk, ARFID appears to be under-recognized in youth with chronic health conditions [15].

## 4. Risk Factors for Binge-Eating Disorder

No studies have specifically evaluated the prevalence of binge-eating disorder among youth with chronic health conditions. However, it stands to reason that youth with chronic health conditions would be at high risk for developing binge-eating disorder, since risk factors for binge-eating disorder include poor self-esteem [61,62,63,64], poor body image [61,63,65], depression [62,66,67,68], and anxiety [62,67,68], all of which are highly prevalent in this population. 

Binge-eating disorder is certainly strongly associated with many chronic diseases. For example, among youth with chronic health conditions that include the metabolic syndrome, diabetes, hypertension, and/or dyslipidemia, there is a strong association with binge-eating disorder [21,22,23,24]. Interestingly, there appears to be a relationship between binge-eating disorder and metabolic dysfunction that is independent of the presence of obesity [21,22]. Binge-eating disorder is also associated with gastrointestinal disorders, asthma, menstrual dysfunction, and polycystic ovarian syndrome [22]. The directionality of the relationship between binge-eating disorder and these chronic health conditions has not been well-characterized.

## 5. Illness-Specific Disordered Eating Behaviors

A concerning phenomenon specific to youth with chronic health conditions is illness-specific disordered eating behavior—that is, the manipulation of treatment for one’s illness for the purposes of weight control. These are some of the most dangerous eating disorder behaviors in which youth can engage. The most well-known example of this is intentional insulin omission among patients with type I diabetes for the purposes of inducing weight loss, which was first described by Polonsky et al. [69]. This behavior is commonly referred to as “diabulimia” as insulin omission serves as a method of purging, and is associated with a higher risk of vascular complications and increased mortality [70]. Other examples reported in the literature include intentional underuse of pancreatic-enzyme replacement therapy in patients with cystic fibrosis [71] and intentional increase in levothyroxine dose among patients with thyroid disease [72]. Other examples from the author’s clinical experience include intentional avoidance of anti-nausea medications in patients experiencing vomiting from chemotherapy, as well as intentional omission of steroid treatments to avoid weight gain.

## 6. Early Identification of Eating Disorders

Health care providers who care for youth with chronic health conditions must be aware of the strong relationship between eating disorders and chronic illness. Providers may take the following steps to identify and treat eating disorders and disordered eating behaviors in this vulnerable population. 

### 6.1. Detect Changes in Growth Early and Consider a Broad Differential Diagnosis

In any child or adolescent with a chronic health condition who appears to be losing weight, providers should consider a broad differential diagnosis. Inadequate control of the underlying condition (i.e., flare or recurrence of disease, inadequate adherence to treatment plans) must always be a consideration, but disordered eating should also be considered. Even in the presence of active organic disease, disordered eating may be playing a role. All contributors to malnutrition must be identified and treated simultaneously. Any unexpected deviation from the child’s normal growth trajectory should prompt non-stigmatizing questions about changes in the youth’s patterns of nutrition and physical activity, including disordered eating behaviors. 

### 6.2. Universally Screen Adolescents and Young Adults for Body Image Concerns and Disordered Eating

All youth should be screened for poor body image and engagement in disordered eating behaviors regardless of weight or health status [73], but it is particularly important in youth with chronic health conditions. Screening is essential because earlier diagnosis and treatment of eating disorders is associated with an improved prognosis [74,75].

Screening can be routinely completed at annual health surveillance visits. It can be helpful to ask the youth about disordered eating and body image without caregivers present, after explaining the limits of confidentiality. The provider may open the conversation by asking if the youth has any concerns about their weight, shape, or appearance. If the youth indicates that they have body image concerns, the provider may explore whether the youth has engaged in disordered eating behaviors in response to their thoughts, particularly dietary restriction (which may alternate with binge-eating), skipping meals, exercising for weight loss, or more dangerous weight control behaviors (e.g., aggressive dietary restriction, self-induced vomiting, diet pill or laxative abuse). Learning that the child is engaging in dangerous weight control behaviors would require the provider to break confidentiality. 

The SCOFF questionnaire is a five-item validated tool that providers may use to screen youth for disordered eating behaviors [76], but it has limitations; it can be useful to screen for anorexia nervosa and bulimia nervosa, but does not adequately screen for other DSM-5 eating disorders [77]. The 26-item Eating Assessment Tool (EAT-26) is a longer screening tool than the SCOFF, but it is one of the most widely used standardized assessments of eating disorder symptoms and has been validated in adolescents [78] and children as young as 8 years old [79].

There are few eating disorder screening questionnaires that are specific to individuals with chronic health conditions. One notable example is the Diabetes Eating Problem Survey—Revised (DEPS-R), a 16-item questionnaire validated in youth with type I diabetes, which includes questions related to insulin manipulation and intentional achievement of hyperglycemia and ketosis for the purposes of weight loss [80]. The development of other condition-specific eating disorder screens could allow for the earlier detection of other dangerous illness-related eating disorder behaviors. 

### 6.3. If Poor Body Image or Disordered Eating Behaviors Are Identified, Advise the Patient about Disease-Specific Risks and refer Promptly to a Multidisciplinary Team of Providers with Expertise in Eating Disorders

As discussed earlier, eating disorder behaviors among youth with chronic health conditions are associated with increased morbidity and mortality. However, many youth with chronic health conditions are unaware of the additional risks conferred by their underlying condition when they engage in risky behaviors. It is critical for health care providers to educate adolescents with chronic health conditions about how they have more to lose than their peers if they engage in these behaviors. Youth need to understand the disease-specific risks they face if they engage in disordered eating behaviors, and how high the stakes really are. 

Prompt referral to a multidisciplinary team of providers with experience in the treatment of eating disorders, including mental health and certified nutrition professionals, can greatly support the adolescent and their caregivers in managing both the eating disorder and the underlying chronic health condition [81].

### 6.4. Partner with All of the Adults in a Young Person’s Life to Facilitate the Earliest Possible Detection of Poor Body Image and Disordered Eating Behaviors

Physicians may be the last to know that a young person is battling an eating disorder and/or poor body image. Youth may be uncomfortable disclosing disordered eating behaviors or distress related to body image when they are in a doctor’s office. However, other adults who play meaningful roles in the child’s life, including parents and other caregivers, mental health providers, teachers and school counselors, athletic trainers and coaches, and cultural and spiritual leaders may be better-positioned to recognize a developing eating disorder. They observe the child in a variety of contexts (including meal times) and know the child well enough to notice changes in eating and exercise behaviors as well as social or emotional changes. Health care providers who care for youth with chronic health conditions can educate their caregivers about their elevated risk for disordered eating, and request their assistance in monitoring the child for emerging concerns and quickly seeking appropriate support. They can also advocate for educators, coaches, and community groups to have access to training that increases their awareness of eating disorders and opportunities for early intervention.

## 7. Preventing Eating Disorders among Youth with Chronic Health Conditions

### 7.1. Screen Frequently for Associated Mental Health Concerns and Provide Appropriate Resources

Given the burden of mental illness in this population, which increases both the risk of developing an eating disorder and eating disorder severity, frequent screening for depression, anxiety, poor self-esteem, and poor coping is critical. Screening can be performed by administering validated questionnaires such as the Patient Health Questionnaire 9 (PHQ-9) [82] depression screen, the Generalized Anxiety Disorder 7 (GAD-7) [83] questionnaire, or the Screen for Child Anxiety-Related Emotional Disorders (SCARED) [84] questionnaire. In this population, providers could also consider using questionnaires to evaluate illness-related stress and quality of life, such as the Pediatric Quality of Life (PedsQL) questionnaire [85]. Some disease-specific questionnaires to assess health-related quality of life are also available, such as:

- IMPACT-III [86]—a health-related quality of life questionnaire specific to inflammatory bowel disease, which assesses four domains: general well-being, social functioning, emotional functioning, and body image.
- The Diabetes Quality of Life (DQOL) questionnaire [87]—available in a shortened 15-item form that strongly correlates with patient satisfaction with their diabetes control, self-care behaviors, and health-related worry about the future.

Referring to a variety of available options for integrated, school-based, and/or community-based mental health support can reduce barriers to accessing this essential care. In-person and online support groups, ideally those that are facilitated, can foster positive social connections and increase resiliency. 

### 7.2. In the Course of Clinical Care, Only Recommend Dietary Restrictions That Are Evidence-Based and Necessary

Many chronic health conditions require dietary restrictions as the primary pillar of management (e.g., a gluten-free diet in celiac disease). However, “trials” of exclusionary diets (e.g., gluten-free, dairy-free) to treat symptoms or improve disease control can act as a catalyst for more general restrictive eating [88]. Even medically necessary dietary restrictions such as the avoidance of food allergens are strongly associated with eating disorders [42]. Essentially, “restriction begets restriction”; vulnerable youth may be unable to limit their dietary restriction to the medical concern at hand. Therefore, the benefits of any dietary intervention should be weighed against the potential risks of precipitating disordered eating behaviors. 

For example, dietary protein restriction has traditionally been used in patients with chronic kidney disease to slow progression to end-stage renal failure. However, adolescents with chronic kidney disease and hypertension who have received dietary recommendations for medical reasons also show a higher prevalence of disordered eating, comparable to adolescents with type I diabetes [89]. It is therefore worth examining how much these dietary recommendations truly benefit this population. A systematic review demonstrated that low-protein diets were not associated with any statistically significant improvement in renal deaths or progression to end-stage renal disease among children with chronic kidney disease [90]. Recently, the Pediatric Renal Nutrition Taskforce published a clinical practice guideline stating that the restriction of dietary protein should generally be avoided in children with chronic kidney disease; rather, they suggest using urea levels, phosphate load, and acidosis as markers to determine whether specific children might benefit from protein restriction [91]. Similar approaches that use risk-based stratification rather than generalized recommendations for dietary restriction can reduce the risks of both malnutrition and disordered eating. 

### 7.3. Engage Caregivers to Encourage a Healthy Relationship with Food from the Earliest Stages of Childhood

Providers can give anticipatory guidance to parents and caregivers about creating a culture of healthy and happy eating in their homes. This is helpful for all children and adolescents, but particularly those with risk factors for disordered eating. Providers can introduce concepts of the Total Diet Approach [92], which is recommended for people of all ages to prevent overweight, underweight, and disordered eating behaviors [73,81,92]. Incorporating the Total Diet Approach into nutrition counseling for youth with chronic health conditions could include the following:

- Even if some foods need to be limited or avoided in the context of one’s chronic illness, nutrients from all of the major food groups (calcium-rich foods, dietary fats, proteins, carbohydrates, fruits, and vegetables) are still needed for health. Nutrients from all food groups should be included in adequate relative proportions. As much nutrition as possible should be consumed as real foods rather than dietary supplements.
- Aside from foods that are clearly contraindicated in the context of an underlying condition, all other foods can fit into a healthy dietary pattern. Avoid the dichotomization of foods as “good” or “bad”, and adopt a neutral stance towards all medically safe foods. Vilifying packaged snacks while praising fruits and vegetables is an oversimplification; both a diet comprising mostly potato chips and a diet comprising mostly broccoli, at the expense of other food groups, would be associated with negative health effects. Sufficient variety in one’s overall pattern of eating is more important than the individual foods consumed.
- Ensure that enough energy is consumed to permit normal growth and development and to support regular enjoyable physical activity.

Providers cannot overemphasize that parents and caregivers are critical nutritional role models to their children, whether they are aware of it or not. Caregivers and parents who themselves engage in dieting, frequent weight talk, and/or weight-related criticism or teasing are more likely to have children who struggle with disordered eating and weight disorders [93,94,95,96]. Family meals should be encouraged as they reduce the risk of disordered eating among children and adolescents [97,98,99,100]. It is helpful to remind families that flexibility, spontaneity, and enjoyment of social and celebration eating are important components of a healthy nutrition pattern. 

### 7.4. In Youth with Chronic Conditions That Are Associated with or Exacerbated by Obesity, Take Particular Precautions to Minimize Weight Stigma and Keep Recommendations Behaviorally Based

It is well-known that many chronic health conditions faced by children and adolescents are associated with the presence of obesity, such as type II diabetes, dyslipidemia, polycystic ovarian syndrome, intracranial hypertension, hypertension, chronic kidney disease, chronic liver disease, musculoskeletal conditions, and obstructive sleep apnea (summarized in [101]). Children may have underlying conditions that increase the risk of obesity and its associated comorbidities, including genetic disorders, endocrine disorders, and inborn errors of metabolism [102,103,104]. Obesity is more prevalent among youth with autism, ADHD, and behavioral health disorders [105,106,107,108]. Children may require treatment with medications that are associated with weight gain, such as systemic steroids, anti-epileptic medications, or psychotropic drugs [109]. 

In these patients, it is understandable that providers would want to encourage their patients to consider weight management an important treatment goal. However, a provider recommending weight loss is an independent risk factor for increased eating disorder risk [110]. Youth with higher weights are known to have a higher prevalence of disordered eating behaviors than their peers [111,112,113,114,115,116,117,118,119,120]. It is also becoming increasingly common for youth with a history of overweight to be diagnosed with eating disorders following attempts to lose weight [121,122,123]. The DSM-5 now includes the diagnosis “atypical anorexia nervosa”, which refers to individuals who have experienced significant weight loss, dysmorphic body image, and fear of weight gain, but present at a normal or even high weight [1], often due to a history of being overweight [124,125]. Although youth with atypical anorexia do not appear underweight, they experience physical and psychological complications that are just as severe as those with traditional anorexia nervosa [121,126,127,128,129,130,131]. 

Youth with both chronic health conditions and higher weights should be presumed to be at particularly high risk for eating disorders and would benefit from regular screening for disordered eating behaviors and poor body image [132]. If a provider determines that a patient’s elevated weight status or pattern of nutrition and/or physical activity poses risks to their health, the following evidence-based suggestions can facilitate healthy changes without increasing their risk for developing an eating disorder:

- Keep the focus on health rather than weight [133]. Avoid prescribing weight loss or setting weight targets. Explain that the goal is not to achieve a weight loss goal, but rather to improve specific parameters of health such as blood pressure, pulmonary function, or lipid levels.
- Make behaviorally based recommendations [73] with a focus on balanced patterns of nutrition consistent with the Total Diet Approach [92], enjoyable physical activity, and limitations on screen time.
- Do not praise rapid weight loss, which is associated with a higher risk of medical complications [126,134] and should raise the provider’s concern for potentially dangerous disordered eating behaviors. Instead, offer praise and encouragement for adopting healthful patterns of nutrition and physical activity.
- Be conscientious of language used when discussing weight status [135,136,137,138,139]. Not only is it critical to avoid obviously derogatory terms such as “heavy”, “chubby”, or “fat”, but even the words “overweight” and “obese” are often perceived negatively and may damage rapport with your patient if used. More neutral terms such as “higher weight” or “higher BMI” are preferred.
- Bias against higher-weight individuals is extremely prevalent among individual health care providers and systematically in health care settings [140,141,142]. Unfortunately, higher-weight youth who experience weight stigma tend to gain more weight over time, perpetuating a vicious cycle [138,143,144,145,146]. Experiencing weight stigma is associated with the worsening of other physical markers including elevations in blood pressure, cortisol, C-reactive protein, and hemoglobin A1c [147,148]. Youth who experience weight bias also exhibit higher rates of psychological complications including depression, anxiety, suicidality, eating disorder behaviors, poor body image, and substance abuse [138,144,145,149]. Providers must avoid the assumption that weight status is entirely based on behavior or willpower, when in fact there are multiple social and genetic determinants of weight status [101,150]. Reflecting on one’s implicit bias as a provider and consciously changing one’s approach to be health-focused, rather than weight-focused, has the potential to significantly improve outcomes [137,141].

### 7.5. Promote Media Literacy and Act as a Trustworthy Source of Nutritional Information for Youth with Chronic Health Conditions

Unfortunately, there is ample evidence that exposure to social media content regarding nutrition and exercise is likely to worsen body image and increase the risk of eating disorders and/or disordered eating behaviors [151,152,153,154]. Adolescents with chronic health conditions are more likely than their peers to seek health information, including information on nutrition and exercise, on the Internet and social media [155,156]. Even if they may recognize that a social media site is not necessarily accurate, adolescents with chronic health conditions tend to prefer social media sites over more scientific sources, as they find them more relatable and value the ability to share information and exchange support with peers who have similar health concerns [156,157]. Adolescents are also most likely to evaluate the reliability of information presented on social media by the number of “likes” or views that it has generated [157]. Youth are, therefore, highly vulnerable to adopting fad diets and social media nutritional trends without scientific basis, and are exposed to unrealistic ideals of body weight and shape. 

Providers have an opportunity to provide anticipatory guidance to youth about how to critically appraise social media messaging around weight and shape, nutrition, and exercise in order to enjoy the benefits of social connection without damaging their self-esteem or body image. Ensuring a consistently open and non-judgmental approach with youth, including the provision of confidential spaces in which they can ask their own questions, can encourage them to approach providers to “fact-check” the nutritional messages to which they are exposed. 

## 8. Conclusions

It is critical to recognize that youth with chronic health conditions are at higher risk for all types of eating disorders while facing greater morbidity and mortality if eating disorders develop. However, health care providers can offer support for modifiable risk factors and provide appropriate anticipatory guidance to patients and caregivers from early childhood onwards. A thoughtful approach to prevention can facilitate a healthy relationship with food and positive self-concept. If eating disorders do develop, prompt recognition and referral to treatment is essential for improving prognosis. Future research directions could explore the prevalence and characteristics of binge-eating disorder among youth with chronic health conditions, which has been relatively unexplored; the development of more disease-specific screening tools to facilitate earlier detection of eating disorders; risk-based stratification to minimize the extent to which dietary interventions need to be used for disease management; and the development of effective media literacy education specific to youth with chronic health conditions. 

## Data Availability

No new data were created or analyzed in this study. Data sharing is not applicable to this article.
